# Prymnesins: Toxic Metabolites of the Golden Alga, *Prymnesium parvum* Carter (Haptophyta)

**DOI:** 10.3390/md8030678

**Published:** 2010-03-16

**Authors:** Schonna R. Manning, John W. La Claire

**Affiliations:** Section of MCD Biology, The University of Texas at Austin, 1 University Station, A6700, Austin, Texas 78712, USA

**Keywords:** harmful algal blooms (HAB), ichthyotoxins, prymnesins, Prymnesium parvum, polyketides

## Abstract

Increasingly over the past century, seasonal fish kills associated with toxic blooms of *Prymnesium parvum* have devastated aquaculture and native fish, shellfish, and mollusk populations worldwide. Protracted blooms of *P. parvum* can result in major disturbances to the local ecology and extensive monetary losses. Toxicity of this alga is attributed to a collection of compounds known as prymnesins, which exhibit potent cytotoxic, hemolytic, neurotoxic and ichthyotoxic effects. These secondary metabolites are especially damaging to gill-breathing organisms and they are believed to interact directly with plasma membranes, compromising integrity by permitting ion leakage. Several factors appear to function in the activation and potency of prymnesins including salinity, pH, ion availability, and growth phase. Prymnesins may function as defense compounds to prevent herbivory and some investigations suggest that they have allelopathic roles. Since the last extensive review was published, two prymnesins have been chemically characterized and ongoing investigations are aimed at the purification and analysis of numerous other toxic metabolites from this alga. More information is needed to unravel the mechanisms of prymnesin synthesis and the significance of these metabolites. Such work should greatly improve our limited understanding of the physiology and biochemistry of *P. parvum* and how to mitigate its blooms.

## 1. Introduction

Several species of *Prymnesium* Massart are known to synthesize noxious chemicals as measured by their effects on erythrocytes and gill-breathing organisms (Table 1) [1–9]. This organism is especially notorious for seasonal harmful algal blooms (HAB) wherein cell densities increase rapidly with the accompanying presence of potent ichthyotoxins [3,10]. Persistent HAB involving *Prymnesium* can seriously threaten local wildlife, resulting in tremendous losses to the ecology and the economy of the affected regions [2,11]. Due to the breadth of its distribution and the frequency of toxic events, the majority of studies have implicated *P. parvum* and *P. parvum* f*. patelliferum*, which appear to be conspecific and may well represent alternate stages in a haploid-diploid life cycle [12]. The toxic principles of *P. parvum* f. *patelliferum* are similar in their effects and it is possible that they may be identical, or at least closely-related, compounds to those we review here [5,13]. The present review focuses on the toxic principles isolated from *P. parvum*, with exceptions noted.

**Figure 1:**
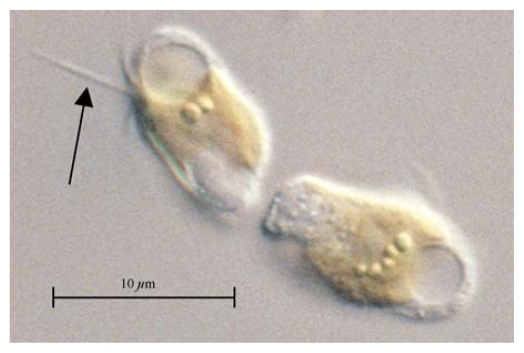
Light micrograph of two *P. parvum* cells (UTEX 2797). The haptonema (arrow) is shown projecting between the two flagella in the upper cell.

*P. parvum* is unicellular, ranging in size from 8–15 μm in length by 4–10 μm wide and having two flagella of roughly equal length positioned at the anterior end (Figure 1) [14–16]. These oblong cells possess two prominent ochre-colored chloroplasts positioned on either side of a centrally-located nucleus. Plastids of these golden (chromist) algae contain chlorophylls-*a*, -*c*, and an abundance of carotenoids [17–19]. A threadlike organelle, the haptonema, projects between the flagella and this structure is a unifying characteristic of the phylum [20]. This structure can vary greatly in length and in function between species; it is very short and non-coiling in *P. parvum* [14]. Proposed uses for the haptonema include it being a sensory device, which is used to attach to substrates or to capture and aggregate prey [21,22]. The haptonema is secretory in species of the related *Chrysochromulina* where glycolipid compounds are released from a region at the base of the organelle [21]. Structurally, the haptonema of *P. parvum* closely resemble those of *Chrysochromulina*, but the secretory attributes have not been demonstrated to our knowledge.

**Figure 2:**
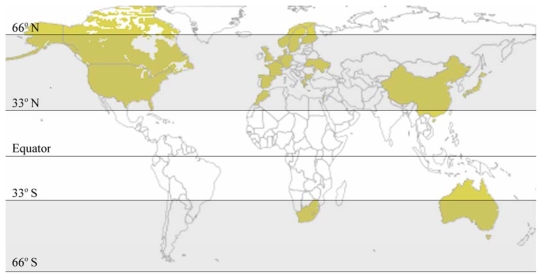
Global distribution of *P. parvum* by country where reported. Most blooms occur in temperate and subtropical zones. Adapted from [2,3,26], and others.

*P. parvum* was first identified by Liebert and Deerns [23] in association with a bloom event in Denmark that reported diseased and dying fish. This alga was later confirmed with another toxic bloom [24]. However, one report suggested that *P. parvum* may have been responsible for earlier fish kills in the Baltic Sea during the late 1800’s [25]. The alga has since been extensively documented as being associated with seasonal toxic blooms and mass mortality events in aquaculture ponds and in native populations of gill-breathing animals. Recurring HAB of *P. parvum* are a nuisance on several continents (Figure 2). The majority of recorded blooms tend to be isolated to cooler waters located in the subtropical and temperate zones between the Tropic of Cancer and Arctic Circle and between the Tropic of Capricorn and Antarctic Circle. HAB of *P. parvum* often form in brackish estuarine waters, but a large number of blooms are known to occur in mainland fresh water reservoirs [27,28]. It is believed that this organism is principally of marine origin [29]. Although reports of *P. parvum*-related fish kills in freshwater reservoirs are growing in number, how this marine organism “invaded” freshwater reservoirs is presently unknown. Proposed vectors of transfer include contaminated bilge water, bird guano and encystment [11,15].

## 2. Prymnesins

Toxin-based investigations with *P. parvum* and other toxic prymnesiophytes have been ongoing for 90 years, and there are several reviews available (e.g., [3,26]). However, a major synthesis of the literature on prymnesins themselves has not been conducted in over 25 years [30–33]. Since last reviewed, two of these compounds have been isolated and chemically characterized [34–37], and phytochemical work indicates the existence of other toxic metabolites from this alga that are under study in various laboratories (e.g., [38,39]). Also, we now know much more about the ecology, molecular biology, and secondary metabolism of this alga that warrant further consideration in this context [40–43]. Surprisingly little is yet known regarding the molecular mechanisms of prymnesin synthesis, the mode of its transport, or the biological relevance of toxin production in this alga.

### 2.1. Isolation and Characterization of Prymnesins

The actual number of different substances that comprise the “prymnesins” is not presently known, but their broad range of biological activities support the notion that extracts from cells and from cell-free supernatants are composed of a complex and diverse mixture of toxic metabolites [30–32,44–47]. For example, Ulitzur and Shilo [48] isolated 6 hemolytic fractions that were potentially chemically distinct from a hemolysin extracted by Kozakai *et al.* [49] and the toxic compound isolated by Paster [39]. Discrepancies between published investigations are likely due to the techniques used for the extraction of prymnesins, resulting in the isolation of unique sets of compounds that separate differently based on their affinity for different solvent(s). Therefore, caution must be exercised when interpreting data and comparing studies. Variations in toxicity reported for different extracts may well be attributed to the extraction methods resulting in extracts containing only a subset of all the possible toxic substances and activities.

Prymnesins have been characterized as glycolipids, galactolipids, proteolipids and lipid-carbohydrate compounds [32,39,48–50]. Crude extractions have been performed with both whole cells and culture filtrates of *P. parvum* and many different approaches have been taken to separate the toxic principles. All of these methods are founded on the differential extraction of prymnesins using an organic mobile phase [32,47]. As a rule, the toxic fractions have been found to be the most soluble in polar organic solvents, and they demonstrate low affinity (and extremely low solubility) in highly non-polar organics. Toxins can be extracted, albeit very slowly, from cells using aqueous solutions; however, extracts from cells are greater than three times more concentrated when extracted with an organic solvent [32]. Shilo and Rosenberger [46] reported that prymnesins were not retained by either anion or cation exchange resins. However, Yariv and Hestrin [31] were able to obtain a concentrated sample of prymnesins by passing culture filtrates over precipitated Mg(OH)_2_, which suggests that some prymnesins demonstrate an affinity for this basic adsorbant material.

Regarding the extraction of prymnesins from whole cells, cells are typically washed several times with cold acetone [34,35,39,46,48]. Shilo and Rosenberger [46] found that prymnesins are not soluble in acetone, which makes this an ideal organic solvent to eliminate interfering compounds including chlorophylls and accessory pigments. Afterwards, polar organics such as methanol, ethanol and/or *n*-propanol are usually employed to extract prymnesins from the cell remains [34,35,39,46,51]. In some cases, multiple rounds of precipitation (e.g., using diethyl ether) are utilized to separate the toxic fractions; further purification of the target compounds can be achieved using column chromatography and/or additional rounds of partitioning between biphasic solvents (e.g., *n-*butanol) [32,39]. Prymnesin extracts have also been prepared from whole algal cell suspensions by liquid-liquid chromatography using a CHCl_3_:MeOH phase system, wherein the toxins are extracted into the lower phase that consists of a suspension of CHCl_3_/MeOH [48,52,53]. It is reported that prymnesins are most stable when they are stored at cooler temperatures as a dry powder; similarly, these extracts are more soluble in organic solvents than in water, and are less labile under acidic conditions near pH 5 [32,54].

**Figure 3:**
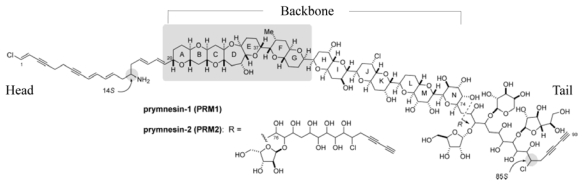
Structures of prym1 and prym2 (inset). Synthesis studies have confirmed the absolute conformation of the ring series A-K and the sinistral (S) chiral centers at carbons 14 and 85. Reproduced with minor modifications with permission from American Chemical Society [37].

It wasn’t until the mid-1990’s that we had the first glimpse of what two of these compounds actually look like. Prymnesin-1 (prym1) and prymnesin-2 (prym2) were meticulously isolated from whole algal cells by a group in Japan and these were the first toxic metabolites to be chemically characterized from any isolate of *P. parvum* using modern analytical methods (Figure 3) [34,35]. Purified prym1 and prym2 were shown to be potent polyketides with ichthyotoxic and hemolytic activities when present at nanomolar concentrations [34,35]. Prym1 and prym2 are ladder-like, polycyclic ethers that posses several noteworthy features. These complex compounds are remarkable in that they have double and triple carbon-carbon bonds in the unsaturated head and tail regions, an amino group, several chlorines, four 1,6-dioxadecalin units, and an assortment of sugar moieties [34,35]. Structurally, prym1 and prym2 are homologous compounds with a common head and backbone: they differ only in the number and in the type of sugars in the tail region, with prym2 containing a rare L-xylose. Prym1 was shown to be slightly more polar (due to the additional sugar residues) and it elutes ahead of prym2 in reversed-phase C-18 chromatography [34,35].

The structural elucidation of prym1 and prym2 has been especially challenging due to their poor solubility in the deuterated organic solvents used with analytical methods such as proton nuclear magnetic resonance (NMR). However, chemical derivatives (e.g., *N*-acetylation) did demonstrate improved solubility and also provided a quantifiable shift in the mass/charge (m/z) for negative ion fast atom bombardment-mass spectrometry (FAB-MS) spectra [34]. However, chemical structures that have been elucidated from spectral analyses are often inaccurate and reassignments are common due to the complexity of many secondary metabolites [55]. The backbone of prym1, comprised of rings A-N, was characterized using a computational approach [56]. Partial (in some cases, total) synthesis of compounds is also useful to reproduce structures and to determine the correct conformations. Recently, investigators have synthesized truncated ring models of prym1 and prym2. This work has led to the verification of the HI/JK rings, the conformation of the chiral centers at C14 and C85, and the reassignment of the juncture between rings E-F (Figure 3) [36,37].

### 2.2. Putative Synthesis of the Polyketides, Prym1 and Prym2

**Figure 4:**
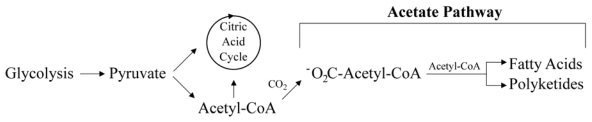
The acetate pathway is responsible for the synthesis of primary and secondary metabolites including fatty acids, polyketides and non-ribosomal peptides. Adapted from Mann [58], with permission from Oxford University Press.

Very little is known about the synthesis of prymnesins *in vivo*. However, it is very likely that prym1 and prym2 are derived from acetate-related metabolism based on what we know about their structures. In other organisms, the acetate pathway is responsible for the generation of fatty acids, as well as numerous secondary products that include polyketides and non-ribosomal peptides (Figure 4) [57,58]. Both primary and secondary metabolites synthesized in this pathway utilize 2-, 3-, and 4-carbon basic skeletal types coupled to coenzyme-A (CoA) [58,59]. Subunits are subsequently combined by successive decarboxylative condensations resulting in chain elongation.

The biosynthesis of polyketides has many features in common with fatty acid synthesis and it uses similar, perhaps some of the same, enzymatic domains. Cane and Walsh [57] described polyketide synthase (PKS) as a modular “megaprotein” possessing a core catalytic domain and auxiliary (carrier) domains. The core domain contains the ketosynthase (KS), acyltransferase (AT) and the acyl carrier protein (ACP)—to which the growing polyketide molecule is tethered via an acyl-*S*-enzyme of the 20 Å long phosphopantetheine arm. The AT domain catalyzes priming of the donor ACP with monomers, and the carbon-carbon bond-forming step is performed by the KS domain. The auxiliary domains encode for ketoreductase (KR), dehydratase (DH), and enoyl reductase (ER). The growing polyketide is handed over from one reactive thiol group to the next by transacetylation reactions. This process is repeated until the ultimate chain length has been achieved, whereupon thioesterase (TE) facilitates the removal of the polyketide by hydrolysis at the distal end of the pantetheine prosthetic group. The individual enzymes function to modify the elongating molecule before adding the next subunit. Chain elongation ceases when all of the “programmed” subunits are incorporated, resulting in a single-chain carbon backbone. Some PKSs also contain enzymes for cyclization (CYC) in addition to other secondary modifications (e.g., methylation) [58,59]. Note that cyclization may occur prior to or after the release of the polyketide from TE.

A minimal PKS operon contains only the gene for KS, whereas diverse PKSs may contain all, parts or none of the respective domain sequences [59,60]. Structurally, prym1 and prym2 resemble other heterocyclic algal toxins produced by Type I PKSs, such as okadaic acid produced by marine diatoms, and brevetoxin and maitotoxin synthesized by the dinoflagellates *Karenia brevis* and *Gambierdiscus toxicus*, respectively [61,62]. These polyether polyketides are non-aromatic, which is characteristic of the Type I modular (non-iterative) PKSs found in bacteria, fungi, animals and some monocotyledonous plants. There is still some debate as to whether PKS associated with dinoflagellates is actually of bacterial origin [63]; in fact, the Type I PKS genes attributed to *Prorocentrum* have been isolated from associated bacteria (*Roseobacter*) [64]. Nevertheless, Type I PKS genes were recently isolated and sequenced from axenic cultures of *P. parvum* [41] and the related haptophyte *Emiliania huxleyi* (Haptophyta) [65], as well as from the green alga *Chlamydomonas reinhardtii* (Chlorophyta) [65].

**Figure 5:**
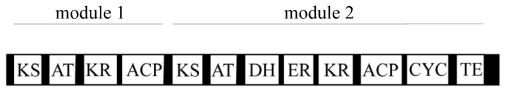
Organization of a putative gene cluster encoding a Type I PKS subunit. The programming of complex polyketides is achieved by discrete modules containing multiple catalytic domains. Adapted from [59,60,66].

In general, Type I PKS genes encode multiple catalytic domains organized into modules and each complete subunit may be composed of 2 or more modules (Figure 5) [64]. Each module contains all functional domains required for exactly one round of chain extension and modification. The complexity and diversity among polyketides and PKSs are a result of mutation, multiple gene duplications, deletions, and horizontal gene transfer [59]. We postulate that these modules might also potentially operate independently of one another in *P. parvum* to generate a diverse array of smaller, yet related polyether molecules, as was demonstrated in mutational analyses using a bacterial PKS [66].

**Figure 6:**
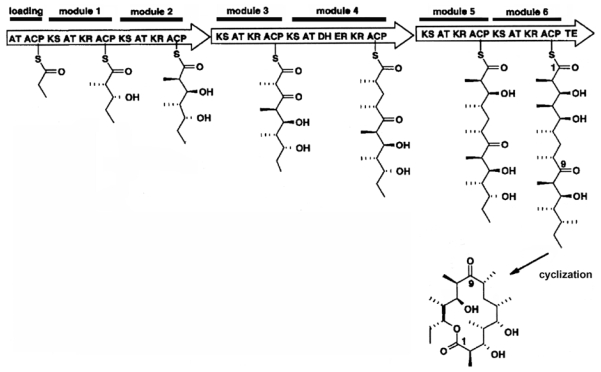
Type I PKS is a modular multiprotein complex. In this example, subunits are added successively by chain elongation tethered to an enzymatic framework. After the ultimate chain length is obtained, the polyketide is released, whereupon it undergoes secondary transformation, such as cyclization. Reproduced with minor modifications with permission from Elsevier [66].

Type I PKS enzymes are analogous to the Type I FAS, and polyketide elongation is performed on an enzymatic framework prearranged into open reading frame modules encoding the mulitmeric protein complex (Figure 6). Because of this, it is likely that polyketide synthesis occurs in the stroma of plastids, which is known to be the case for FAS in green algae [67]. It should be noted that these mechanisms have not yet been elucidated for *P. parvum*. Glycosylation is a common modification of secondary metabolites, and this might be a mechanism for tagging prym1 and prym2 for transport. This process changes the physical and chemical properties of the compounds, their bioactivity and their sub-cellular localization [68]. Post-synthesis modifications (such as glycosylation) probably occur at the Golgi apparatus or endoplasmic reticulum, where many different molecules are tagged and packaged for transport [69]. More information regarding cellular location of polyketide synthesis, storage and transport would be of great importance in determining the biological significance of prymnesins to the alga (see later).

Until recently, there were very few gene sequences available for *P. parvum*. An expressed sequence tag analysis of *P. parvum* identified a number of gene products that may be involved with acetate metabolism and polyketide synthesis [41]. Interestingly, these transcripts have a high sequence similarity to bacterial Type I PKS genes. More specifically, individual Type I PKS genes in *P. parvum* have their nucleotide sequence identities most similar to *Roseovarius* (a bacterium) and *Nostoc* (a cyanobacterium), and one PKS subunit is most closely related to that of a eukaryotic stramenopile [41]. In addition, there are at least 43 different unique genes whose products have been classified as being involved with secondary metabolite synthesis, catabolism and transport, including ACP synthase, oligoketide cyclase, and DH that are potentially relevant to polyketide synthesis. Another 40 unigenes are classified as encoding proteins involved with lipid synthesis and transport, which could also have parallel roles in the manufacture of polyketides. Finally, there are a number of genes expressing vesicle transport-related proteins. Interestingly, the most abundantly expressed gene transcripts have no known sequence homology [41]. More work is needed in this area to determine which of these genes’ products directly participate in the synthesis or transport of prym1, prym2 and other toxic metabolites of *P. parvum*.

The vitamins thiamine (B_1_) and cobalamin (B_12_) are required in culture media and are considered essential for the growth of most known *P. parvum* strains [22,32]. Several genes have been elucidated whose products are related to thiamine and cobalamin metabolism [41]. On the other hand, biotin (B_7_) and pantothenate (B_5_) have not been shown to be essential for growth, yet both vitamins are prosthetic groups essential to the synthesis of primary and secondary metabolites. Acetyl-CoA carboxylase is a biotin-dependent enzyme in the acetate pathway and an essential component in the synthesis of fatty acids and polyketides. Similarly, pantothenate is required for the production of CoA, and in turn ACP. EST analyses indicate that *P. parvum* may possess several genes encoding enzymes involved in these B-vitamin synthetic pathways, and it is therefore likely that *P. parvum* is able to synthesize biotin and pantothenic acid *de novo* [41].

## 3. Modes of Prymnesin Toxicity

Prymnesin extracts are reported to have saponin-like properties that are responsible for a host of deleterious effects including cytotoxicity, neurotoxicity, hemolysis, and ichthyotoxicity [30,31,33]. This broad spectrum of activities is owed in part to their amphipathic and ionophoretic properties [70]. Structurally, prym1 and prym2 are detergent-like compounds possessing both polar and non-polar ends. It should be noted that most toxicity studies have tested crude extracts, which contain toxic fractions as well as other substances. Therefore it is difficult to determine which compound(s) is (are) accountable for any individual biological effect.

Cytotoxicity in the presence of prymnesins is described as possessing two distinct stages. The first response to exposure is cell swelling, which can be measured by the uptake of Trypan Blue dye; the second phase is death/lysis [51]. Cell swelling and lysis can take less than an hour or may take several hours depending on temperature and pH. Elevated pH and temperatures were found to be concomitant with an increase in cell lysis; however, this process could be halted by reversing the culturing conditions [51]. Based on observations with bacteria and membrane preparations, it has been proposed that prymnesins interact directly with specific components of the plasma membrane (e.g., membrane sterols) [32,71]. It is believed that prymnesins form micelles in aqueous solutions (due to their detergent-like properties) and that these aggregates assemble within the plasma membrane of exposed cells to form negatively-charged pores that are permselective to cations [31,50]. Moreover, the prymnesins have been shown to increase membrane conductance, which can be measured as a fluctuation in transmembrane ion concentrations [50,72]. Prymnesin toxicity is known to be dose-dependent and it responds in a linear manner when analyzing log prymnesin concentration *vs.* log membrane conductance.

Prymnesin extracts also possess potent neurological toxicity where it is thought that they interfere with the re-uptake of neurotransmitters at the post-neuronal synapse. Parnas and Abbott [73] demonstrated that this mechanism is carried out at the myoneural junction, where prymnesins inhibit acetylcholine and interfere with calcium signaling. Shilo [74] reported that an intravenous injection of prymnesins stops heart muscle by rapid depolarization and by altering the membrane permeability to calcium ions. In another study, an injection of prymnesin evoked muscle contractions, which was followed by the reversal of the acetylcholine-induced contractions and paralysis [32,75]. An intravenous injection of prymnesins resulted in a sudden drop in blood pressure and respiratory arrest [76]. Meldahl and Fonnum [77,78] discovered that prymnesins from *P. parvum* f. *patelliferum* inhibit the sodium-dependent uptake of L-glutamate and γ-aminobutyric acid, and they enhance the calcium-dependent release of acetylcholine. In accordance, Mariussen *et al*. [79] found that prymnesins were responsible for the calcium-dependent release of glutamine at brain synaptosomes.

Prymnesins are also renowned for their potent erythrolytic capacity and prym1 and prym2 are reported to be 5,000 times more hemolytic than a plant-based saponin [62,80–82]. Crude prymnesin extracts can be isolated from either culture supernatants or cells, however cellular extracts exhibit a higher degree of hemolytic activity [31,47]. Prymnesins isolated from culture media yielded approximately 6700 hemolytic units (H.U.) per mg; by comparison, there was nearly six times this concentration found in cells, representing roughly 40,000 H.U. per mg [32].

The ichthyotoxic effects of prymnesins are by far the most noticeable, due to the frequency of blooms and distress caused by extensive fish kills. Many species of fish are documented as being sensitive to these compounds, as well as many amphibians, mollusks and crustaceans [33]. Prymnesins are noted as being especially lethal to gill-breathing organisms and they can be fatal at nanomolar concentrations [11,52,74,82]. After *Gambusia* were treated with prymnesins in addition to co-factors, their gills became darkly-stained with Trypan Blue within 5 min [1]. Interestingly, Shilo and Aschner [83] found that tadpoles (*Bufo*) were resistant to the effects of prymnesins shortly after metamorphosis, indicating that prymnesins affected tadpoles during the gill-breathing stage only.

Exogenous prymnesins are not documented as being harmful to mammals or mature, lung-breathing amphibians [32]; toxic waters mistakenly used for irrigation and rinsing vegetables had no deleterious effects [53].

## 4. Variables Affecting the Presence and Toxicity of Prymnesins

The presence of prymnesins differs with nutrient availability, salinity, light, temperature, pH, as well as growth stage of the alga [5,11,30,31,39,42,54,84,85]. Moreover, it has been demonstrated that actual prymnesin toxicity varies in dynamic physical and chemical conditions [28,46]. This is not unusual with toxin-producing microalgae. It is well known that growth conditions greatly affect the synthesis of secondary metabolites, and these compounds often increase in the presence of limiting conditions [86,87]. Interestingly, the intensity and range of noxious effects are also dependent on the activation of prymnesins in the presence of specific co-factors [88,89]. The ratio of hemolytic to ichthyotoxic activity is also variable and dependent on certain environmental factors; the hemolytic principle is not always accompanied by toxicity to fish [46,90]. These studies clearly illustrate that toxin synthesis and activation are likely very complicated processes that are potentially synergistic in response to environmental stimuli. As such, this may account for differences in observed toxicity under differing environmental and growth conditions.

### 4.1. Nutrient Availability

*P. parvum* can thrive in a wide range of physical conditions, but nutrient availability has been shown to greatly influence HAB and toxin formation in this organism. Agricultural effluents and eutrophication of waters are commonly implicated in a growth surge in *P. parvum* populations [11,91,92]. High nitrogen (in the form of nitrate or nitrite) and phosphorus (phosphate) concentrations support the rapid growth and formation of blooms that subsequently result in an imbalance of N and P sources. These limiting conditions ultimately slow the growth of *P. parvum*, which is usually accompanied by an increase in toxicity (as noted later) [5,30,93]. Mesocosm experiments over the course of five years indicated a reduction in toxicity in nutrient-replete conditions [43]. These observations suggest that extracellular prymnesins decrease appreciably when growth and nutrient conditions are favorable; therefore, it has been proposed that prymnesin toxicity can be controlled by nutrient manipulation [94]. However, a decrease in exotoxins may also be concomitant with an increase in intracellular toxins (see later).

Phosphorus and (to a lesser degree) nitrogen availability are probably among the most widely-studied factors related to prymnesin toxicity. Shilo [74] was the first to demonstrate elevated toxicity of *P. parvum* in association with low phosphorus conditions, and this trend has been confirmed in subsequent studies [5,77,82,85,95]. Shilo [30] reported a 10- to 20-fold increase in the amount of intracellular prymnesins when grown in P-limited conditions; a rise in toxin concentration was also documented for culture supernatants. Johansson and Granéli [95] analyzed the effects of N and P levels on cell density, chemical composition and toxicity of *P. parvum*, being the first to show increased toxicity under N-limiting conditions. Hemolytic activity was observed regardless of the nutrient conditions, so they proposed that cell stress (resulting in lysis and death?) was the cause for increased toxicity rather than the direct involvement of either N or P in toxin synthesis *per se* [96]. This is also in agreement with analyses confirming increased toxicity in senescing/dying cultures of *P. parvum* (see later).

The addition of a carbon source has also been shown to augment toxicity in populations of *P. parvum*. Dark-incubated cultures were able to grow in glycerol-supplemented media [84], and the addition of glycerol has been demonstrated to cause an increase in toxicity [97]. We postulate that this increased toxicity might be correlated to a surge in glycolytic activity from the exogenous carbon source. This would provide precursor molecules for the acetate pathway that might be used for polyketide synthesis. Conversely, glycerol and glucose have been shown to interfere with the formation of secondary metabolites in some organisms [86]. It is obvious that more information is needed to determine the roles of various nutrients in prymnesin toxicity and possible inhibitory pathways.

Beyond being photosynthetic, *P. parvum* is also known to phagocytose bacteria and other photosynthetic algae, which may provide exogenous N, P and carbon sources in times of nutrient limitation [98,99]. Legrand *et al*. [94] demonstrated that *P. parvum* f. *patelliferum* utilizes various sources of phosphate including inorganic, dissolved, organic and particulate forms; similarly, *P. parvum* has been shown to utilize ammonia, methionine and ethionine as alternative N-sources [22,100]. Recent findings by Carvalho and Granéli [101] indicate that *P. parvum* is truly mixotrophic and therefore feeds heterotrophically under both nutrient-limiting conditions and in nutrient-replete cultures. Interestingly, bacterial suspensions of *Proteus vulgaris* and *Bacillus subtilis* decreased toxicity by 50% in one hour [83], suggesting that these bacteria may be capable of metabolizing at least some of the toxins [102].

### 4.2. Salinity

Due to the predominance of blooms in low salinity and brackish water systems, many studies have examined the effects of salinity on prymnesin toxicity. The broad osmotic tolerances of *P. parvum* have been well-documented [5,22,97,103]. In most cases, *P. parvum* has been recorded in highly-mineralized freshwaters between 3 and 8 ppt (e.g., [28]); some assert that *P. parvum* can grow almost anywhere, in salinities ranging from 1 to 45 ppt [22,40,103]. However, low salinity conditions have been shown to have a negative effect on growth rates of *P. parvum*. Baker *et al*. [104] illustrated that there is a decrease in growth rate concurrent with a decrease in salinity, even when grown in nutrient-sufficient conditions. Interestingly, Dickson and Kirst [27] postulated that the broad osmotic tolerance of *P. parvum* is related to their ability to synthesize a range of compatible solutes coupled to a partial exclusion of toxic ions. The fact that gene transcripts encoding various ion transporters were among the most highly-expressed ones in log-phase cultures of *P. parvum*, may also help to explain how this alga is able to handle such a broad range of salinities [41].

Specific ions may play a role in prymnesin toxicity, but these assessments are limited and conflicting data have been reported. Paster [32] noted that the optimum NaCl concentration for growth and toxin production in *P. parvum* is between 0.3–5% (=3–50 ppt) (see also [1]) Salt begins to inhibit toxicity at levels greater that 5–12% NaCl [32]. This is not surprising as this represents salt concentrations of 50–120 ppt, exceeding two to four times the salinity of open ocean seawater. Paster [32] found elevated ichthyotoxicity for cultures grown in 5% (0.75 ppt) artificial seawater (ASW that contained ~15 g/L salts) and the lowest toxicities are documented for cultures grown in 30% (4.5 ppt) ASW. Interestingly, there appeared to be an absence of ichthyotoxicity in salinities >30% seawater. However, a lack of extracellular bioactivity is not directly related to intracellular toxin production, as there are still large quantities of toxins able to be extracted from the cells themselves [32,76,105]. Shilo and Rosenberger [46] reported that the hemolytic property of prymnesins was inversely related to salinity with the highest hemolysis observed for cultures grown in 1% salt (=10 ppt). In contrast, Larsen and Bryant [40] found no significant differences in toxicity for *P. parvum* when grown at different salinities, which they felt could indicate metabolic differences among different strains of the alga. Since a decrease in toxicity in the culture media was reported to coincide with an increase in intracellular toxins [76], we conclude that both the cells and the supernatants need to be carefully analyzed in order to determine any relationships between salinity and toxicity.

### 4.3. Light

Light is a factor that is often associated with prymnesin toxicity, but published observations are somewhat inconsistent. *P. parvum* has been documented as naturally occurring in moderate-to-low light conditions, but some isolates have shown a preference for (and grow better with) higher illumination [40,106,107]. However, where *P. parvum* actually exists in the environment may be somewhat dependent on local community structure, which may also play a role in the stratification and distribution of *P. parvum* in the water column.

Many studies have shown that both light quality and light intensity are physical properties that are essential for the synthesis and activity of prymnesins [28,47,74]. On the other hand, some have concluded that light is not necessary for toxin production in *P. parvum* [108]. Dark-grown cultures reportedly have a reduced amount of intracellular toxins, which supports the latter claim; but an increase in toxin concentration was detected later in the stationary phase of culture [89]. This finding is in agreement with growth studies that observed differences in toxicity and it suggests that toxic compounds are accumulating in the cells steadily throughout the exponential growth curve (see later). Since *P. parvum* is mixotrophic and can feed in the absence of light, these accounts suggest that light may not be necessary as long as there is sufficient food to provide carbon skeletons for prymnesin synthesis.

Interestingly, cultures that were grown under constant illumination demonstrated no ichthyotoxic activity, but did retain the hemolytic activity [106]. It was postulated that the ichthyotoxic principles may either be inactivated or they may degrade more rapidly than the compounds responsible for hemolysis [45]; alternatively, it is possible that multiple factors are responsible for hemolysis, some of which remain to be characterized. In accordance, exotoxins were inactivated after 90 min when treated with UV and visible wavelengths of light (255 nm and 400–520 nm, respectively) [107,109]; however, intracellular toxins were not affected, suggesting that these compounds are being protected by some mechanism or they may be stored in vesicles [97].

### 4.4. Temperature

*P. parvum* is eurythermal, being capable of growing at temperatures ranging from 2–30 °C [22,28,39,83]. This has made it difficult to determine whether ambient temperature plays a significant role in prymnesin presence or toxicity. Globally, recurring HAB of *P. parvum* do not always occur at the same time of the year. Blooms in the southern United States appear throughout the fall and winter months [26]; in China, blooms form in the late spring and early fall [28]; and a summer bloom in Finland was characterized as having water surface temperatures around 20–25° C [53].

Similarly, optimum temperatures for growth and toxicity vary greatly among different geographic isolates of the alga. The optimum temperature of a strain from Denmark was around 26 °C, and growth was limited by temperatures near 10 °C [11]. In contrast, Larsen and Bryant [40] found that most (but not all) of the strains they studied had maximum growth rates at 15 °C. Given the broad temperature tolerances of *P. parvum*, there are no clear-cut correlations between specific temperatures and toxicity [30].

### 4.5. pH

The various modes of prymnesin toxicity are also restricted to different pH ranges, with hemolysis and ichthyotoxicity highest during acidic and alkaline pH conditions, respectively. Prymnesins are also acid- and base-labile, which means that they are only functional under certain pH conditions [52]. Shilo and Rosenberger [46] demonstrated that an increase in pH to alkaline levels eliminated the hemolytic activity, but did not appear to affect ichthyotoxicity. In fact, these authors state that toxicity in fish requires a pH > 7.0, which is in agreement with the observations that toxic *P. parvum* occurs in Chinese waters ranging from pH 7.2 to 9.3 [28]. Ulitzur and Shilo [88] reported that activation of the ichthyotoxic property is dependent on cationic activation at a pH greater than 8.0 with maximum toxicity observed at pH 9.0 (see below). Conversely, hemolysis can occur in conditions as low as pH 5, with decreasing hemolytic activity with increasing alkalinity. These studies show that pH can greatly affect the range, nature and intensity of prymnesins’ toxic properties, which also suggests that numerous (and probably some unknown) compounds may be at work here.

### 4.6. Co-factors

The primary reason why the presence of prymnesins and actual ichthyotoxicity are not necessarily related is because co-factors are required to “activate” prymnesins [5,30,31,85]. Furthermore, the type of activator determines the extent and severity of the toxic effects [89]. Toxicity to fish is greatly enhanced by the presence of Na^+^, Ca^2+^, and Mg^2+^, as well as by antibiotics (e.g., streptomycin) and polyamines (e.g., spermine) [1,30,88,89,105]. Shilo and Rosenberger [46] established that divalent cations were more effective than monovalent ones in promoting ichthyotoxicity. These ion interactions may play a role in the aggregation, complexation and structural conformation of prymnesin molecules (as are common with polyether ionophores, e.g., the monensin-Na^+^ complex [110]), which may in turn affect the ability of prymnesins to interact with specific components of the plasma membrane [1,70].

### 4.7. Biotic Factors

Toxicity in this alga and in culture filtrates also varies with growth phase, *i.e.*, lag phase, exponential phase and stationary phase cultures, in addition to the abiotic and community interactions mentioned [30,83]. While Paster [32] found a significant positive correlation between the concentration of *P. parvum* cells and the degree of exotoxins, some have suggested that factors necessary for growth and toxicity are regulated by different parameters [74,111]. In fact, Reich and Aschner [10] noted that concentration of prymnesins in the surrounding media is not always related to the intracellular concentrations of prymnesins. Extensive mortalities have been observed with low cell concentrations, and dense blooms of *P. parvum* have been documented as having no toxic effects [74,83]. Guo *et al*. [28] confirmed that the appearance of extracellular toxins from *P. parvum* has been shown to be independent of cell density, with the two parameters having no clearly-defined relationship.

A reduction in ichthyotoxicity is typically observed during periods of rapid growth (e.g., exponential phase) and the effects of prymnesins are most obvious when populations of *P. parvum* reach limiting conditions (e.g., stationary phase) [28]. Similarly, Houdan *et al*. [4] reported that *P. parvum* cultures were less toxic to cultures of the brachiopod crustacean *Artemia* while in the exponential, growing phase. During the stationary phase (which happens to correspond to the highest cell concentrations and limiting conditions), they found complete mortality of *Artemia* nauplii. This is not surprising since secondary metabolism increases during periods when balanced growth has ceased [87]. This is often the case with eutrophication leading to surge in algal growth, which is subsequently followed by nutrient imbalance and limiting conditions (e.g., [93,95]).

Berman [111] followed the total endo- and exotoxin content in growing cultures. In this study, it was noted that the concentrations of both hemolytic and ichthyotoxic compounds increased with culture age on a per-cell basis, implying that these compounds might be synthesized constitutively and accumulate as a result. In agreement, Paster [32] reported that prymnesins are synthesized throughout the alga’s life cycle, and are only released after cell death and disintegration of the cell components. This also lends more support to prymnesins serving a possible intrinsic function, because it would seem unlikely that cells would store waste products long-term (see later). Superficially, it would appear that there is a difference in the presence of endo- and exotoxins, but more information is needed regarding the mechanism of release to make further conclusions on the roles of these compounds.

Differences among published studies could be attributed to the methods of extraction, how cells and cell-free supernatants were being compared, how toxicity was quantified, the presence of cofactors, examining cultured *vs*. natural samples, differing geographic isolates, *etc.*

## 5. Biological Relevance of Prymnesins

It has been suggested that under certain conditions, the production of prymnesins may contribute in a number of ways toward improving the growth of the alga, or alternatively they may behave primarily as defensive compounds as are so many other secondary metabolites [112,113]. Prymnesins have been largely depicted as being extracellular (exo-) toxins, but the precise mechanisms of how these substances are released into the environment are not presently known [31,109]. Given their presence in water samples and in culture media, it is postulated that prymnesins could be either secreted, excreted or released into the environment as a result of cell lysis and death (or some combination thereof) [83,95,114]. Physiologically, these terms imply very different processes for the externalization of prymnesins, as well as the biological role(s) of prymnesins and the health of the alga.

Prymnesins can be found in great abundance in the cells themselves as compared to culture filtrates, and this would lead one to believe that prymnesins are fulfilling some internal function; it is clearly not favorable to synthesize an abundance of energetically-expensive compounds without imparting some benefit to the organism producing the substance(s) in question [31,32,46,47]. Many secondary metabolites have intracellular roles as endotoxins, signaling compounds or storage products [87], and we postulate that prymnesins may have similar functions.

The defensive properties of prymnesins have been well-defined, and it is obvious that the presence of prymnesins imparts numerous benefits to populations of *P. parvum*. Prymnesins have been shown to deter herbivores and reduce grazing, which also affects the feeding habits and viability of predator species [115–117]. For example, copepod feeding was lower in the presence of *P. parvum* f. *patelliferum* and the animals preferred non-toxic algae if given the choice [115,118,119]. Although it is not always clear whether lowered feeding is a result of the presence of exotoxins or the avoidance of noxious algae, these studies support the notion that toxin production may aid in the growth and survival of *P. parvum* by making the alga a less palatable option.

Production of prymnesins may also confer *P. parvum* with the ability to capture prey organisms. Prymnesins are toxic to other algae and certain bacteria, and it was proposed that prymnesin toxicity was a mechanism for paralyzing prey prior to ingestion [38]. Later observations confirmed that prymnesins effectively paralyzed or killed prey organisms, which were rapidly phagocytosed thereafter by encroaching *P. parvum* cells [117,120,121]. Skovgaard and Hansen [114] pointed out that this is a functional advantage because *P. parvum* is otherwise unable to catch prey due to its short, stiff haptonema, as compared to many other haptophytes. Furthermore, if the haptonema is indeed secretory in this alga, it would be interesting to know the constituents of this secreted material to determine if they are related in any way to toxicity of *P. parvum*.

Prymnesins as exotoxins also have allelopathic effects by the elimination of competing algal species [31,116,122,123]. In fact, Houdan *et al*. [4] noted that competing algae lost the ability to swim in the presence of prymnesins. It has been suggested that prymnesins, as allelochemicals, may play a role in its ecological strategy due to the modest growth rate of *P. parvum* [103,124,125]. However, it cannot be stated unequivocally that prymnesins are allelochemicals until a mechanism of delivery is shown to correlate directly with any observed effects [126].

Finally, if prymnesins are released as a process of excretion, this would suggest that these substances are mere waste products. Prymnesins are also very likely to be released into the environment as a result of cell lysis. Future studies aimed at sub-cellular localization of the prymnesins and ones identifying potential metabolic and secretory pathways will be necessary to unravel the mechanisms by which these toxins wind up in the surrounding medium. This information would also be of significance in deciphering the incentives behind synthesizing prymnesins as well as in clarifying any of their intrinsic and extrinsic functions.

## 6. Detection of Prymnesins

Presently, there are no methods available for the specific detection of individual prymnesins themselves. The structural elucidation of the complex prym1 and prym2 molecules was a significant advancement in the study of this organism that has opened the door for the study of other toxic metabolites in *P. parvum*. Prym1 and prym2 were chemically characterized by positive-mode ESI-LC/MS and NMR [34,35]. While, these and related analytical methods are highly-sensitive and capable of validating both mass and structural features, generating procedures for mass spectra can be very complex, expensive, and time-consuming. Toxin-containing fractions must also be highly-enriched and contain few interfering substances for confident detection and quantification. Moreover, there are no standards presently available for prym1, prym2, or for crude extracts of prymnesins. Consequently, such spectroscopic methods are not practical for prymnesin identification in natural or cultured samples. The chemical isolation of individual prymnesins has proved especially challenging, and new methods will need to be developed for the specific *in vitro* detection of toxic metabolites from this alga.

Bioassay-based methods are widely used to detect the effects of prymnesins. These have been developed to quantify the various bioactivities of prymnesin extracts incorporating erythrocyte, crustacean, and fish model systems [32,40,127]. Due to the different modes of toxicity, ideally more than one type of assay should be employed to quantify the individual effects of toxin extracts [52,82]. It is also important to keep in mind that the various toxin activities are dependent on co-factors or electrolytes in the assay media, which may not be reflective of toxicity in native/environmental conditions.

The hemolytic assay is considered very sensitive and requires 100-fold less prymnesins per unit volume than ichthyotoxicity-based assays [32]. Rabbit erythrocytes have been used for a number of analyses [32,39,45], while others have utilized other animal and fish as sources of red blood cells [31]. Hemolytic activity of prymnesins is dependent upon the presence of electrolytes in the assay medium, and it is estimated that approximately 5000 prymnesin molecules are needed per erythrocyte to initiate lysis [32]. The strain of *P. parvum* also appears to contribute to different results with this assay. For example, the cell concentration of *P. parvum* that elicits 50% hemolysis was approximately 10,000 cells/mL compared to 50,000 cells/mL for *P. parvum* f. *patelliferum* [52]. Eschbach *et al*. [127] used carp erythrocytes to test toxicity of whole cell extracts and compared results with saponin-based standards. In their assessment, hemolytic activity was recorded as a function of absorbance at 414 nm (from released hemoglobin) and the extent of hemolysis was sigmoidal as a function of prymnesin concentration. The hemolytic concentration necessary to elicit 50% hemolysis (HC_50_) for purified prym2 was 10.5 nM; hemolytic activity was unaffected by Ca^2+^ but was affected by erythrocyte origin [128].

Toxicity of prymnesins can also be measured by their effects on crustaceans (e.g., *Artemia*) and fish (e.g., *Gambusia*) [88]. Both strain and growth phase of the alga may result in differences in observed toxicity. The lethal concentration of *P. parvum* cells leading to 50% *Artemia* mortality was recorded as being approximately ~1,000 cells/mL, whereas ~2,700 cells/mL was necessary when using a strain of *P. parvum* f. *patelliferum* [52]. With respect to growth phase of *P. parvum*, less than 1.0 × 10^5^ stationary-phase cells/mL were required for 50% *Artemia* mortality *vs*. 5.0 × 10^5^ exponential-phase cells/mL [4]. Clearly, the effects of prymnesins on crustaceans are not easily comparable and therefore may not represent equal measures of toxicity depending on growth phase and algal strain. The lethal concentration of purified prym2 with 50% fish mortality (LC_50_) is 300 nM without any co-factor, and 3 nM with the addition of 2.0 mM Ca^2+^ [128]. The addition of a co-factor obviously promotes a 100-fold increase in toxicity in this system, which may be the cause of heightened toxicity in mineral-rich environments.

Alternatively, as a substitute for measuring toxins, there are several methods available for the specific detection of *P. parvum* cells themselves. Traditionally, *P. parvum* identity is confirmed by microscopy, but many cell and molecular techniques are now available in addition to morphology-based identifications. Oligonucleotide (rRNA) probes were designed for the detection of *Prymnesium* utilizing dot-blot hybridization and fluorescence *in situ* hybridization [129,130]. In addition, solid-phase cytometry methods are available for the identification of *P. parvum* using fluorescently-labeled species-specific probes and monoclonal antibodies [131–133]. Conventional polymerase chain reaction (PCR)-based detection and quantification was made possible for several isolates of *P. parvum* using species-specific oligonucleotide primers [134]. These techniques are capable of detecting several geographic isolates of *P. parvum*, as well as some isolates of the conspecific, *P. parvum* f*. patelliferum*. The most recently developed assays utilize real-time, qPCR technology for the detection and quantification of *P. parvum* [135,136]. These methods are particularly useful for the early detection of *P. parvum* when cell concentrations and toxic effects are below detectable limits.

## 7. Conclusions and Future Prospects

HAB events of the last century have greatly increased the awareness of prymnesins, toxic principles produced by the golden alga, *P. parvum*. It is obvious that many abiotic and biotic factors appear to be correlated with toxin presence within cells and in the surrounding medium, and overall prymnesin activity. Still it is difficult to generate models correlating prymnesin synthesis, toxicity and bloom formation partly because published reports are on disparate strains of *P. parvum* and toxin-based analyses are at times conflicting. Extreme differences can exist in toxin content and in toxin composition between different geographic isolates, and these differences are closely tied to the local environmental conditions and community ecology [87]. Therefore, it is believed that many of these discrepancies can be attributed to inherent qualities of the various geographic strains for this widely-distributed alga [40]. Moreover, the broad spectrum of toxic effects raises the question of how many compounds are actually responsible for toxicity in this alga, and we postulate that the type and number of prymnesins may expand in the future and may also differ among geographic isolates of *P. parvum*.

The inactivation of prymnesins and eradication of *P. parvum* would be of great interest to fish and wildlife biologists working to mitigate HAB. The inactivation of prymnesin toxicity has been observed to occur under certain physicochemical conditions, but many methods are not feasible when balancing healthy fish culture and while retaining beneficial algae. Furthermore, it is difficult to determine which of these many toxic substances are being inactivated or if some of these toxins are merely inactive under the given conditions. Ammonium and copper sulfates are commonly employed for the eradication of *P. parvum* in fish culture ponds [28,89,137], but these chemicals have to be used at levels necessary to kill the alga without severely harming the fish [138]. Gou *et al*. [28] also tested the effectiveness of fertilizers and mud, but found that reducing the salinity to less than 2 ppt effectively suppressed algal growth without compromising the health of the fish. As was noted, *P. parvum* and its ensuing prymnesin toxicity might also be controlled by nutrient manipulation [94]. Finally, the use of flocculant clays has also shown some promise for the mitigation of *P. parvum*, but the clays appear to cause an increase in the presence of prymnesins, which is likely due to the stress and sedimentation from clay treatment [96].

Many variables play into observed differences in prymnesin toxicity, but the central issues lie with the specific extraction technique and the source of the toxin extraction (cells *vs*. culture filtrates). Each extraction technique potentially represents a different subset of toxic substances with variable activities and it is obvious that standard methods of extraction and quantification need to be established for any meaningful comparative purposes. Furthermore, we conclude that environmental parameters need to be established for individual geographic isolates of *P. parvum* so that we may better understand toxicity in this alga. Genome- and chemical-based investigations have presented avenues for probe development and gene expression analyses. The confirmation of the gene products thought to be involved in prymnesin synthesis would be of great interest, since they could potentially be manipulated using environmental controls and mutational analyses [87]. The sub-cellular location of prymnesins’ synthetic pathways, their sequestration and their mode of release into the environment would be of great value in determining the roles of toxin-production in this alga. It is clear that a more complete understanding of the molecular and biochemical triggers of prymnesin synthesis are needed in order to better monitor this and other HAB-forming organisms.

## Figures and Tables

**Table 1:** Species of *Prymnesium* documented as toxic to aquatic life. Adapted from [1–4], and others.

Species	Source
*Prymnesium parvum*	[2,3]
*P. parvum* f. *patelliferum*	[5]
*P. calathiferum*	[6]
*P. faveolatum*	[7]
*P. saltans*	[7–9*]
*P. zebrinum*	[7]

* Organism described with one fish kill may have actually been *P. parvum* [2].
